# Exploring gene editing as a potential therapeutic strategy for hemophilia

**DOI:** 10.3389/fbioe.2026.1727204

**Published:** 2026-02-04

**Authors:** Nishal Kumarasamy, Balaji Balakrishnan

**Affiliations:** Gene Therapy Laboratory, Department of Integrative Biology, School of Bio Sciences and Technology, Vellore Institute of Technology, Vellore, Tamil Nadu, India

**Keywords:** CRISPR/Cas9, gene editing, hemophilia, iPSCs, TALENs, ZFNs

## Abstract

Hemophilia is an inherited bleeding disorder caused by mutations in the *F8* or *F9* gene, leading to a deficiency or dysfunction of coagulation factors VIII or IX. While current treatments, such as factor replacement, extended half-life factors, and gene therapy, have improved patient outcomes, they have limitations such as immunogenicity, transient transgene expression, and the requirement for high vector doses. Gene editing for hemophilia is an emerging approach that aims to provide a permanent cure by editing the mutated gene precisely or targeted integration of coagulation factor cDNA into the host genome for stable expression. This approach involves the use of programmable nucleases (CRISPR/Cas9, TALENs, ZFNs) that induce double-stranded DNA breaks at specific sites, allowing precise correction or targeted transgene integration. This review covers the various editing tools and strategies used for precise gene editing in hemophilia, including approaches such as HDR, NHEJ, base editing, prime editing, *ex vivo* gene editing in iPSCs, and recent LNP-based CRISPR delivery methods for precise editing.

## 1 Introduction

Hemophilia A and B are X-linked recessive bleeding disorders caused by mutations in the blood coagulation factor VIII (FVIII) and factor IX (FIX) genes, respectively. These mutations lead to deficiencies or dysfunction of the corresponding clotting factors, contributing to prolonged bleeding episodes. Severe hemophilia patients (factor activity <1 IU/dL) often suffer from spontaneous bleeding in joints and muscles. Prophylactic or on-demand factor replacement therapy with recombinant factors or plasma-derived clotting factor concentrates has improved patient outcomes. However, they remain expensive, and the patients require frequent infusions. Moreover, a section of the patients develops inhibitors (neutralising antibodies against the infused factors) over time, thus complicating hemophilia treatment (Garagiola et al., 2018; Perrin et al., 2019; Nathwani, 2019). Bispecific antibody therapy (utilizing emicizumab, a FVIIIa mimetic) for hemophilia A and rebalancing therapy (utilizing agents like fitusiran or marstacimab) show promise in restoring hemostasis (Mahlangu et al., 2023; Castaman et al., 2025; Young et al., 2023; Mannucci, 2020). However, the need for factor replacement in severe conditions like trauma or surgery and during breakthrough bleeding cannot be fully eliminated. (Marchesini et al., 2021; Cohen et al., 2023; Gogia et al., 2023).

Gene therapy for hemophilia is an attractive strategy that aims to deliver a functional copy of the clotting factor cDNA, thereby offering a potential cure for the disease (Davidoff and Nathwani, 2016; Coppola et al., 2025). Adeno-associated virus (AAV) vector-based gene therapy for hemophilia has made remarkable progress over the past two decades. Notably, advances in gene therapy for hemophilia B include long-term FIX expression in patients (St. Jude/UCL phase 1/2 trial) (Nathwani et al., 2018). Stable low level of FIX was observed for up to 13 years in hemophilia B patients who received AAV8-FIX (Reiss et al., 2025), while a stable high level of FIX was seen in individuals who received AAV5-FIX Padua variant for more than 5 years (Samelson-Jones Benjamin et al., 2021a). This progress led to the approval of Hemgenix (etranacogene dezaparvovec), an AAV-based gene therapy drug, by the US-FDA and its conditional approval by the European Medicines Agency (EMA) (Heo, 2023). Another AAV vector-based gene therapy, Beqvez (Fidanacogene elaparvovec), developed by Spark Therapeutics and Pfizer, was approved in December 2023 for adults with moderate to severe hemophilia B (Dhillon, 2024). However, concerns remain regarding the cytotoxic T cell responses against AAV transduced hepatocytes and loss of transgene expression in the high-dose group (Costa-Verdera et al., 2023).

Gene therapy for hemophilia A is relatively challenging due to the large size of the FVIII cDNA ∼5 (kb) expression cassette and decline in the transgene expression over time (Samelson-Jones et al., 2024). To overcome AAV packaging constraints, most clinical programs employ a B-domain-deleted FVIII (BDD-FVIII) in which the large central B domain, which is not required for procoagulant activity, is removed. In liver-directed gene therapy for hemophilia A, the first clinical trial employing AAV5-BDD-FVIII-SQ achieved transiently normalised FVIII levels in the first year, which subsequently declined towards the mild hemophilia range during years 2–5 (Pasi et al., 2020; Pasi et al., 2021). However, the highest dose group (6 × 10^13^ vg/kg) achieved a mean FVIII activity of 64 IU/dL at the end of year one (Pasi et al., 2020). Subsequently, another clinical trial employing such high doses (3 × 10^13^ vg/kg) of AAV6-BDD-FVIII resulted in a mean FVIII activity of 42.6 IU/dL at week 52 (Leavitt et al., 2024). This success led to the progression to phase 3 trials and subsequent approval of valoctocogene roxaparvovec (AAV5-BDD-FVIII-SQ) by the US-FDA and conditional approval by the EMA (Samelson-Jones et al., 2024; Blair, 2022).

However, long-term follow-up (6 years post-infusion) revealed a 7-fold decrease in FVIII levels (even with the highest vector doses) from 64 IU/dL at year 1 to 9.8 IU/dL at year 6 (Pasi et al., 2020; Symington et al., 2024). Such an inadequate and decline in transgene expression over time has also been observed in other trials (High et al., 2018; Giermasz et al., 2022). In addition, more participants of hemophilia A trials (85.8%) developed transaminitis compared to hemophilia B trials (16.7%) (Pierce et al., 2024). In the dividing cells of paediatric patients, potential loss of factor expression due to the episomal nature of the transgene is also a concern (High and Roncarolo, 2019). In a few studies, patients who received the vector infusion, also continued factor prophylaxis due to the loss of transgene expression (George et al., 2021; Ozelo et al., 2022).

In contrast, gene editing technologies have emerged as promising tools that potentially offer stable expression of clotting factors associated with hemophilia (Wang et al., 2019). By precisely editing/replacing the mutated loci, these approaches aim to restore functional clotting factor expression. This could be done by using programmable nucleases such as Zinc Finger Nucleases (ZFNs), Transcription activator-like effector nucleases (TALENs), and Clustered Regularly Interspaced Short Palindromic Repeats/Cas9 protein (CRISPR/Cas9) (Robles-Rodríguez et al., 2020). These engineered nucleases create double-stranded breaks (DSBs) at specific genomic loci. The DSBs could then be allowed to repair either by error-prone non-homologous end joining (NHEJ) or by homology-directed repair (HDR) in the presence of an exogenous homologous template.

Several preclinical studies have utilised both HDR for targeted integration of coagulation factors and NHEJ for gene correction, advancing the evaluation of safety and efficacy in hemophilia (Park et al., 2016; Anguela et al., 2013; Alayoubi et al., 2024). Beyond delivering exogenous cDNA, gene editing tools can directly correct pathogenic variants in the endogenous *F8* or *F9* loci or modulate regulatory pathways to improve hemostasis. Base editing has demonstrated precise correction of hemophilia point mutations, restoring FVIII expression (Tonetto et al., 2024). Similarly, in hemophilia B models, CRISPR-mediated insertion of highly active FIX Padua variant, has achieved promising therapeutic levels. Prime editing further broadens the range of correctable mutations by enabling flexible edits without double-stranded breaks (Arruda and Samelson-Jones, 2016; Samelson-Jones BenJ. et al., 2021b). Notably, *in vivo* CRISPR gene editing for hemophilia B has recently entered early-stage human clinical trials (NCT06379789), marking a major milestone towards durable genomic cures (https://clinicaltrials.gov/study/NCT06379789). Although challenges such as off-target effects and delivery efficiency persist, recent studies provide encouraging evidence that gene editing could serve as a potentially curative next-generation treatment for genetic disorders (Zeballos et al., 2021; Paul et al., 2022) This review examines the advantages and limitations of these approaches, as well as their potential for permanent disease correction. We also highlight recent preclinical studies that demonstrate meaningful progress toward developing safe and effective therapies.

## 2 Review methodology

We conducted a literature search on gene-editing strategies for hemophilia using PubMed, Scopus, and Google Scholar, without applying any filters on publication year. Keywords included: Hemophilia, Gene editing, CRISPR, ZFNs, TALENs, Induced pluripotent stem cells, and rebalancing therapy.

All article types- original research, preclinical studies, animal models, clinical trials, and reviews were considered based on titles and abstracts. Studies were included if they addressed gene-editing approaches for hemophilia A or hemophilia B in any model (*in vitro*, *in vivo*, or *ex vivo*). No exclusions were applied for publication year, study design, or experimental system.

Relevant articles were qualitatively analyzed for:

1. Gene-editing platform (CRISPR/Cas9, ZFN, TALEN)
2. Target locus and editing strategy
3. Delivery method (viral/non-viral)
4. Preclinical or clinical outcomes
5. Rebalancing therapy
6. Induced pluripotent stem cells
7. Safety considerations and reported limitations

Findings were synthesized into a narrative overview of current progress and future directions in hemophilia gene editing.

## 3 Genome editing by Zinc Finger Nucleases (ZFN)

ZFNs enable targeted genome editing by creating DSB at specific DNA sites. Each ZFN has two components: a DNA-binding domain and a DNA-cleavage domain. The DNA-binding domain contains zinc finger motifs that can be engineered to recognise chosen DNA sequences. The cleavage domain is the *FokI* restriction endonuclease, which cuts target DNA when two ZFNs dimerize (Kim et al., 1996). When left and right ZFNs are introduced into cells, the *FokI* domains pair up and induce DSBs at the target locus (Carroll, 2011).

**FIGURE 1:**
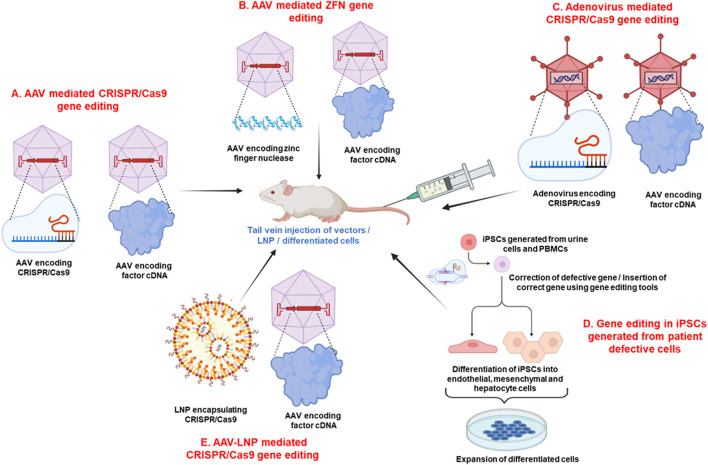
Pre-clinical gene editing strategies for Hemophilia. **(A,B)** AAV-mediated delivery of CRISPR/Cas9/ZFNs and coagulation factor cDNA. **(C)** Adenovirus-mediated delivery of CRISPR/Cas9 and coagulation factor cDNA. **(D)** CRISPR/Cas9/TALEN mediated correction of patient-derived iPSCs and differentiation into therapeutic cell types. **(E)** AAV-LNP combined delivery systems. AAV vectors encoding coagulation factor, LNPs, or cells from all these strategies are administered *via* tail vein injection in the hemophilia mouse model to evaluate restoration of clotting factor expression. Image created using BioRender.

**FIGURE 2:**
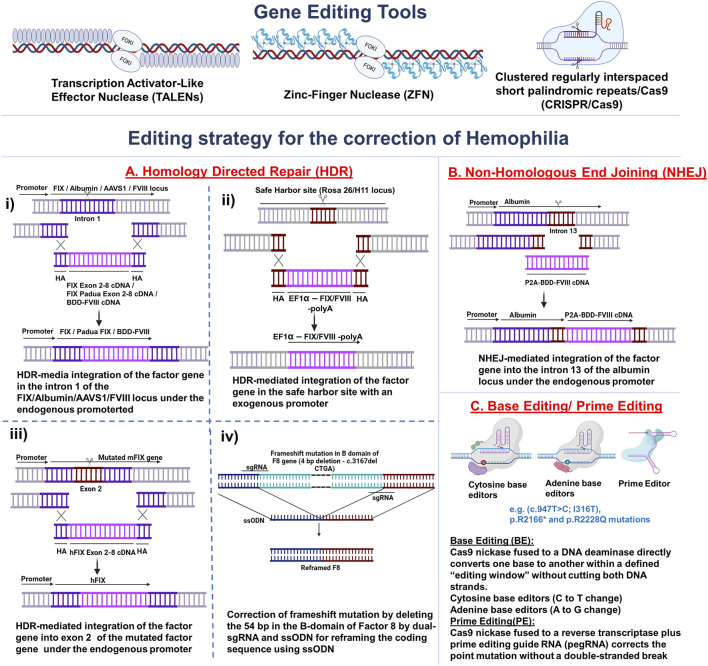
Gene editing tools for Hemophilia. Gene editing tools, such as Transcriptional Activator-Like Effector Nuclease (TALENs), Zinc-Finger Nuclease (ZFN), and Clustered Regularly Interspaced Short Palindromic Repeats/Cas9 (CRISPR/Cas9), are used to correct defective genes or integrate coagulation factor cDNA. **(A)** Homology-directed repair (HDR) mediated gene editing. **(i)** HDR-mediated integration of the factor gene in the intron 1 of the *FIX/Albumin/AAVS1/FVIII* locus under the endogenous promoter; **(ii)** HDR-mediated integration of the factor gene in the safe harbour site *(Rosa 26/H11*) with an exogenous promoter; **(iii)** HDR-mediated integration of the factor gene into exon 2 of the mutated factor gene under the endogenous promoter; **(iv)** Correction of frameshift mutation (4bp deletion – c.3167del CTGA) by deleting the 54 bp in the B-domain of Factor 8 by dual-sgRNA and ssODN for reframing the coding sequence using ssODN, **(B)** Non-Homologous End Joining (NHEJ) mediated integration of the factor gene into the intron 13 of the *albumin* locus under the endogenous promoter; **(C)** Point mutations (c.947T>C; I316T, p.R2166* and p.R2228Q) are corrected by base editing or prime editing. Image created using BioRender.

Early proof-of-concept studies highlighted the therapeutic potential of ZFNs in treating hemophilia Li et al. (2011) demonstrated this in a humanized mouse model carrying an *hF9* mini-gene with a Y155stop mutation. This mutation truncated exons 7 and 8, leading to a lack of circulating coagulating FIX. In their approach, intron 1 of *hF9* was targeted by delivering *F9-* specific ZFNs driven by a liver-specific enhancer and promoter. A promoter-less transgene comprising human *F9* exons 2-8 flanked by splice acceptor and polyadenylation sites was integrated *via* HDR. Both ZFNs and donor templates were delivered using AAV8 vectors: 5 × 10^10^ vg of AAV8-ZFN and 2.5 × 10^11^ vg of AAV8-F9 donor. These were injected intraperitoneally into neonatal hemophilia B mice on day 2 of life (Figures 1, 2). Although the HDR efficiency was modest (1%–3%), treated mice showed circulatory factor IX level of 166–354 ng/mL (3%–7% of normal circulating levels) (Table 1). Importantly, their activated partial thromboplastin time (aPTT) was significantly reduced compared to hemophilia B mice. Long-term stable FIX expression lasting up to 30 weeks was confirmed through partial hepatectomy (Li et al., 2011).

**TABLE 1:** Preclinical studies of Hemophilia.

Gene editing platform	Disorder	Target locus	Animal model	Transgene	Dose	Factor level/activity	References
ZFN	Haem B	*F9*, Intron 1	Neonatal *F9*mut/HB mice	AAV8-ZFN, AAV8-F9 donor	5 × 10^10^ vg, 2.5 × 10^11^ vg	166–354 ng/mL, (3%–7% of normal)	Li et al. (2011)
ZFN	Haem B	*F9*, Intron 1	Adult *hF9*mut/HB mice	AAV8-ZFN, AAV8-F9 donor	1 × 10^11^ vg, 5 × 10^11^ vg	1,146 ± 100ng/mL (23% of normal)	Anguela et al. (2013)
ZFN	Haem B	*Albumin*, Intron 1	Adult C57BL/6 mice	AAV8-ZFN, AAV8-hF9-donor	1 × 10^11^ vg, 5 × 10^11^ vg	>3000ng/mL	Sharma et al. (2015)
ZFN	Haem A	*Albumin*, Intron 1	HA/CD4null	AAV8-ZFN, AAV8-ZFN, AAV8-h*F8*-donor	5 × 10^10^ vg, 5 × 10^10^ vg, 1 × 10^11^ vg	37% ± 5.5% activity of normal	Sharma et al. (2015)
CRISPR/Cas9	Haem B	*F9* Exon 2	Adult *F9* KO mice	AAV8.sgRNA. hFIX co-padua donor, AAV8.Sacas9	5 × 10^12^ GC, 5 × 10^11^ GC	171.6% ± 5.2% of normal activity, 8.2% ± 1.2% of normal protein level	Wang et al. (2019)
CRISPR/Cas9	Haem A	*mAlb* Intron 13	*F8KO*	AAV8-SaCas9 AAV-BDD-F8	Total dose of 6 × 10^11^ vg/kg	13% of normal activity	Chen et al. (2019)
CRISPR/Cas9	Haem B	Intron 1 of *mF9*	*F9* KO mice	AAV8-SaCas9, AAV8-mFIX (exon 2–8)	1 × 10^12^ vg, 3 × 10^12^ vg	FIX:C - 11.7%–39.6%	Ohmori et al. (2017)
CRISPR/Cas9	Haem B	Intron 1 of *albumin*	B6.129P2-*F9* ^*tm1Dws*^/J (8-week-old male)	AAV8.SpCas9 AAV8.sgRNA.donor	9 × 10^10^ GC, 4.5 × 10^11^ GC	40% of normal Factor levels, 162% ± 29% of normal activity	Wang Q. et al. (2020)
CRISPR/Cas9	Haem B	*Apoc3* Intron 1	Humanized mice (B6.Apoc3APOC3)	AAV-CjCas9 and AAV-hF9	(4 × 10^13^ vg/kg for each AAV)	250 ng/μL (HITI),50 ng/mL (AAV-trap)	Lee et al. (2022)
CRISPR/Cas9	Haem B	*ROSA26*	R33Q (Hemophlia B Murine model)	Ad5-EF1α-mFIX, Ad5-cas9-gRNA	7.5 × 10^10^ VP 2.5 × 10^10^ VP	419 ng/mL of mFIX level	Stephens et al. (2019)
CRISPR/Cas9	Haem B	*Serpinc1*	C57BL/6-*F9* ^em1^ (F9^Mut^) mice	AAV8-Bidirectional hF9 LNP-Cas9 mRNA	2 × 10^13^ vg/kg 1.2 mpk	500–700 ng/mL after 20 weeks	Lee et al. (2023)
CRISPR/Cas9	Haem A	*Serpinc1*	C57BL/6.*F8* intron 22 inversion (F8^I22I^) mice	AAV8-BDD-FVIII LNP-Cas9 mRNA	1 × 10^12^ vg/kg 1.5 mpk	25 ng/mL - In liver lysate	Han et al. (2023)

Anguela et al. (2013) extended ZFN-based gene editing to adult hemophilic mice with quiescent livers. They administered 1 × 10^11^ vg of AAV8-ZFN and 5 × 10^11^ vg of AAV8-F9 donor (containing homology arms) into eight-week-old *hF9* mutant mice. At 60 weeks, the animals showed an average hFIX level of 23% of normal (1,146 ± 100 ng/mL), a 5-fold increase compared to the neonatal mice (Table 1). Importantly, sustained FIX expression was observed up to 12 weeks in groups receiving both homology-dependent and independent donor vectors. This finding suggested that NHEJ-mediated integration could support gene correction in quiescent tissues. Off-target activity was minimized by employing AAV-ZFN vectors engineered with an obligate heterodimeric Fok I architecture, incorporating the ELD: KKR mutation in the dimerisation interface to improve specificity (Anguela et al., 2013). These early investigations established ZFNs as the first generation of targeted nucleases, specifically applied in hemophilia models. They provided proof-of-concept, showing that precise genome modification using engineered ZFNs was feasible.

Targeting diseased loci directly is often inefficient due to limited accessibility, low targeting rates and insufficient expression from endogenous promoters. To overcome this limitation, Sharma et al. (2015) selected a safe harbour locus with strong transcriptional activity to integrate coagulation factor genes. They chose the mouse serum albumin locus, inserting integrated BDD-FVIII and FIX (exon 2–8) cDNA into the first intron (Figure 2). This design allowed expression under the albumin promoter with the exon 1-encoded secretory peptide cleaved from the final protein. In hemophilia B/CD4 null mice, administration of 1 × 10^11^ vg of AAV8-ZFN and 5 × 10^11^ vg of AAV8-hF9-donor (containing exons 2–8 with splice acceptor and poly A signal) produced circulating FIX levels above 3,000 ng/mL, which remained stable for over a year (Table 1). Interestingly, only 0.5% of hybrid mAlb-hF9 mRNA relative to wild-type mAlb was detected, showing that minimal editing in hepatocytes was sufficient to achieve normal FIX levels in the blood circulation (Sharma et al., 2015). A similar approach was applied to hemophilia A mice (HA/CD4 null). Two AAV8-ZFN vectors (5 × 10^10^ vg each) plus an AAV8-hF8-donor (1 × 10^11^ vg, with 600 bp homology arms) achieved hFVIII activity 37% ± 5.5% of normal (Table 1). In both hemophilia A and B models, ZFN-mediated integration restored haemostasis. However, off-target analysis revealed INDELS at 11 out of 40 predicted sites (Sharma et al., 2015). Despite these promising preclinical results, translation to humans has been challenging. In a phase I clinical trial (NCT02695160) by Sangamo Therapeutics, a patient received 5 × 10^13^ vg/kg of three rAAV6 vectors encoding left and right ZFN plus a promoter-less transgene for albumin intron 1 integration. At day 60, FIX activity increased from 1 IU/dL to 17 IU/dL, but the patient still required factor concentrates. Furthermore, two episodes of bleeding were observed. (Harmatz et al., 2022). Nevertheless, long-term follow-up of the patient to study the safety of ZFN *in vivo* is ongoing (NCT04628871).

Apart from *in vivo* gene therapy, *ex vivo* integration of FVIII into autologous cells is another promising option for treating hemophilia A. Towards this, Sivalingam et al. (2016), chose primary cell types such as human umbilical cord-lining epithelial cells (CLECs), dermal fibroblasts, bone marrow- and adipose tissue-derived stromal cells, and integrated FVIII transgene into *AAVS1* safe harbour site. Among these, CLECs produced the highest and most stable FVIII levels. After puromycin selection, CLECs secreted about 2,131 ± 17 mU/10^6^ cells over 24 h, measured 37 days post-electroporation with ZFN and donor DNA plasmids. Deep sequencing showed a low frequency of INDELS, and no off-target integration was detected in CLECs (Sivalingam et al., 2016).

Mesenchymal stem cells (MSCs) were also evaluated for *ex vivo* gene transfer because they are less immunogenic and engraft better post administration (Ullah et al., 2019; Bu et al., 2024). Li et al. (2017) reported targeted integration of *F9* at the *AAVS1* site in human bone marrow-derived (hMSCs). They nucleofected MSCs with a transgene construct (AAVS1-AcGFP1-hFIX, 5 μg) and ZFN plasmids (0.5 μg each of ZFN-L and ZFN-R). Edited MSCs showed a significantly higher FIX expression compared to control cells. When four clones of these edited MSCs were transplanted into Kunming mice, anti-hFIX antibody levels were detected at 5.3, 7.05, 3.05, and 6.15 BU/mL confirming *in vivo* hFIX expression (Li et al., 2017).

Further, David et al. (2023) expanded the range of target cells by using ZFN/AAV6 to insert FIX R338L Padua variant into B cells. The transgene was inserted either at the *AAVS1* site or the *T-cell receptor α constant* (*TRAC*) locus. FIX production was higher when inserted into the TRAC locus, which correlated with greater targeted integration efficiency (*r* ^2^ = 0.7 for TRAC vs. *r* ^2^ = 0.1 for *AAVS1*). B cells were chosen because they can differentiate into plasmablasts and long- lived plasms cells that reside in bone marrow. *In vitro* and *in vivo* experiments showed strong FIX activity (15% of normal FIX activity) along with immunoglobulin expression. The immunoglobulin production confirms that the engineered B cells successfully engrafted in NOD/SCID/Gamma (NSG) mice (David et al., 2023).

ZFNs have also been utilized to study and correct structural variants. Lee et al. (2012) developed a ZFN-based method to induce structural variations (SVs), such as duplications and inversions, to study the potential for flip-flopping of the inverted DNA to the original state in the human genome. They successfully inverted a 140 kbp segment of the FVIII gene in HEK293T/17 cells, mimicking a hemophilia A-associated inversion. Using ZFNs, they were able to revert the segment back to its normal state. Duplication and inversion frequencies ranged from 0.01% to 5%. This approach provides a useful model for studying chromosomal rearrangements and offers potential for correcting genetic defects (Lee et al., 2012).

These studies show that ZFNs offer clear strengths, such as compact design for AAV delivery, precise DNA targeting, and durable gene integration. At the same time, challenges remain, including risks of INDELS at off target sites, modest editing efficiency in hepatocytes, and the need for high vector doses. Overall, while ZFNs laid the groundwork for *in vivo* gene correction in hemophilia, further improvements in delivery, specificity, and long-term safety are required for clinical translation.

## 4 Genome editing by transcription activator-like effector nucleases (TALENS)

TALENs are genome editing tools composed of two parts: a TALE protein from *Xanthomonas* bacteria and a DNA-cleaving domain from the *FokI* restriction enzyme. A typical TALEN system includes a DNA-binding domain, the *FokI* nuclease, an N-terminal nuclear localization signal (NLS), and a flexible linker. The DNA-binding domain is linked to the *Fok1* catalytic domain, and when *Fok1* forms a dimer, it cuts the DNA to create a double-stranded break. Each TALE repeat, giving TALENS precise targeting ability. This differs from zinc finger proteins which bind to three nucleotides at once. After cleavage, cells repair the DNA break through either NHEJ or HDR (Boch et al., 2009; Miller et al., 2011; Gaj et al., 2013).

**FIGURE 3:**
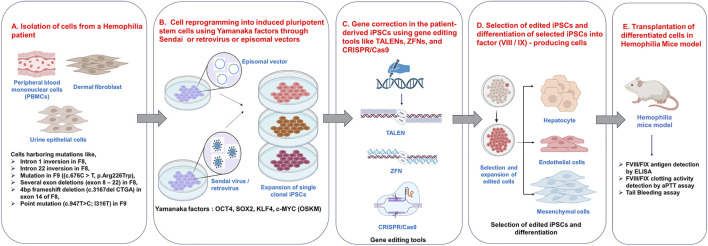
Patient-Derived iPSC-Based Gene Correction and Cell Differentiation Strategy for Hemophilia. **(A)** Isolating cells such as dermal fibroblasts, urine epithelial cells, PBMNCs from hemophilia patients harbouring mutations. **(B)** Reprogramming them into induced pluripotent stem cells (iPSCs) using Yamanaka factor like OCT4, SOX2, KLF4, c-MYC (OSKM) using viral vectors or through episomal vector delivery, **(C)** Editing these cells with gene editing tools like ZFNs, TALENs and CRISPR/Cas9. **(D)** Selection and Differentiation of the edited cells into endothelial cells, mesenchymal cells, or hepatocytes and **(E)** Differentiated cells are evaluated for antigen level and activity or transplanted into hemophilia mouse models to restore factor production and assess the therapeutic potential of the transplanted cells. Image created using BioRender.

In 2014, Park et al. (2014) used TALENS to re-create a 140 kbp chromosomal inversion in the FVIII gene within induced pluripotent stem cells (iPSCs). This inversion mimicked the intron 22 inversion commonly seen in hemophilia A patients. The iPSCs were derived from human dermal fibroblasts (HDFs) (Figure 3). TALEN plasmids targeted intron 22 of the *F8* gene and its extragenic copy, producing the inversion through NHEJ or nonallelic homologous recombination (NAHR). As a result, these iPSCs lost *F8* mRNA expression. Importantly, the same TALENS were able to reverse the inversion, restoring *F8* mRNA. When the corrected iPSCs were differentiated into endothelial cells, FVIII expression was significantly observed compared to unedited cells (Park et al., 2014).

**FIGURE 4:**
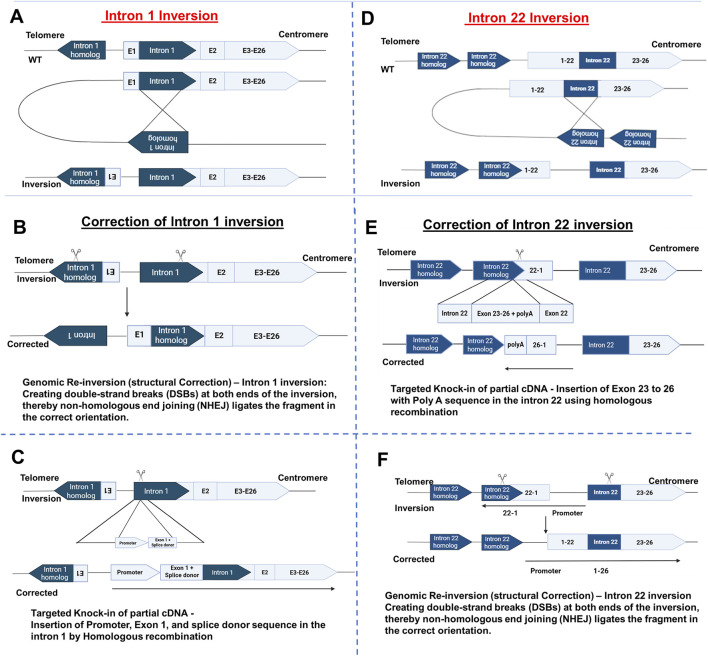
Schematic representation of the intron 1 and 22 inversion causing Hemophilia A. **(A)** Intron 1 inversion. **(B)** Correction of Intron 1 inversion by applying strategies like Genomic re-inversion - Creating DSBs at both ends of the inversion, thereby NHEJ ligates the fragment in the correct orientation. **(C)** Targeted Knock-in of partial cDNA - Insertion of Promoter, Exon 1, and splice donor sequence in the intron 1 by Homologous recombination. **(D)** Intron 22 inversion. **(E)** Correction of Intron 1 inversion by applying strategies like Targeted Knock-in of partial cDNA - Insertion of Exon 23 to 26 with Poly A sequence in the intron 22 using homologous recombination and **(F)** Genomic Re-inversion (structural Correction) of Intron 22 inversion - Creating double-strand breaks (DSBs) at both ends of the inversion, thereby non-homologous end joining (NHEJ) ligates the fragment in the correct orientation. Image created using BioRender.

Wu et al. (2016) applied a similar strategy to iPSCs derived from hemophilia A patients carrying the intron 22 inversion. Using TALENS, they inserted a 627 bp coding sequence (exons 23–26 plus a poly A signal) into the exon 22-intron 22 junction of *F8 via* HDR strategy (Figure 4). This corrected the inversion remarkably with high efficiency (62.5%). FVIII expression was restored in endothelial cells and mesenchymal stem cells derived from the corrected iPSCs. For example, endothelial cells produced 1.12 ± 0.44 ng/10^6^ cells (clone 17-9) and 0.57 ± 0.22 ng/10^6^ cells (clone 11-10). Mesenchymal stem cells produced lower amounts [0.20 ± 0.06 ng/10^6^ cells (clone 17-9) and 0.16 ± 0.08 ng/10^6^ cells (clone 11-10)]. FVIII activity was higher in cells from the corrected iPSCs compared to unedited ones. However, a much larger number of cells would be required to reach therapeutic FVIII levels (Wu et al., 2016).

Building on earlier strategies, Pang et al. (2016), derived iPSCs from hemophilia A patients carrying both intron 1 and intron 22 inversions. This study employed TALENickases, a refined version of TALENs that introduce single-strand nicks rather than double-strand breaks. This approach promotes HDR while avoiding activation of NHEJ pathway. Using TALENickases, the functional FVIII ORF was integrated into the ribosomal DNA (rDNA) locus, which exists in multiple copies. The rDNA arrays, located on the short arm of the ten acrocentric chromosomes, exhibit high recombinational activity. Importantly, the gain or loss of rDNA copies does not cause abnormal phenotypes. The stability and inheritance of this locus, combined with its recombinational activity, make it a suitable site for therapeutic gene integration. Upon differentiation of iPSCs into endothelial cells, FVIII expression was significantly observed by immunocytochemistry (Pang et al., 2016).

Qiu et al. (2021) extended these findings by comparing three TALEN engineered iPSC lines: (i) *in situ* corrected hemophilia A iPSCs (HA-iPSCs, clone 17-9) (44). (ii) BDD-F8-iPSCs (clone T-7-iPSCs), in which the BDD *F8* gene was targeted to the rDNA locus (Pang et al., 2016), and (iii) normal hiPSCs (DYR0100, ATCC). These iPSCs were differentiated into MSCs (17-9-iMSCs, T-7-iMSCs, hiMSCs) and intravenously administered to hemophilia A mice. Stable FVIII activity was observed for up to 4 weeks: 10.32% in mice treated with 17-9-iMSCs, 12.91% with T-7-iMSCs and 9.96% with hiMSCs, compared to 4.78% in untreated hemophilia A mice. Phenotypic correction was confirmed in the *F8*-corrected iMSC groups (derived from *in situ* corrected HA-iPSCs and BDD-F8-iPSCs) using the tail-bleeding assay (Qiu et al., 2021). Collectively, these studies highlight the strengths of TALENs, including their high specificity, capacity for complex chromosomal rearrangements, and adaptability to safe-harbour integration strategies. Nevertheless, several limitations temper the translational potential of TALEN-based therapies. FVIII expression levels in corrected cells remain below the threshold required for clinical efficacy, necessitating transplantation of large cell numbers to approximate therapeutic activity. Moreover, long-term persistence and safety remain to be established *in vivo*. Technical challenges also persist, as TALEN design and assembly are more labour-intensive compared to other engineered nucleases and the risk of off-target cleavage, although reduced by TALENickase refinements, cannot be fully excluded.

## 5 Genome editing by CRISPR/Cas9

CRISPR/Cas9 is a revolutionary genome editing technology that allows precise changes to DNA in specific cell types. Derived from a bacterial defence system, it consists of two components: the Cas9 nuclease and a guide RNA (gRNA) (Davis et al., 2023). The gRNA directs Cas9 to a target DNA sequence, where Cas9 induces a double-strand break. The cell repairs this break either by NHEJ, which often produces small insertions or deletions, or by HDR, when a donor template is available.

Zinc Finger Nucleases (ZFN) were previously used for gene editing in hemophilia A and B, but off-target cleavage caused significant cytotoxicity. Toxicity varied with the location and frequency of ZFN binding sites (Cornu et al., 2008). Because of these limitations, subsequent studies used CRISPR/Cas9, which is simpler and more efficient. In integration-based therapies, selecting the target site demands precision and careful consideration. The selected site must not interfere with the regular operation of other genes. Most CRISPR-based gene editing trials for hemophilia have focussed on integrating functional factor cDNA into defined loci to restore expression. The main strategies are outlined below:

### 5.1 Editing/integration in the coagulation factor locus

The earliest demonstration of CRISPR/Cas9-based gene editing for hemophilia was reported by Guan et al. (2016). This study identified a novel missense mutation (Y371D) in the *F9* gene in a hemophilia B family. Hemophilia mice carrying this mutation were treated with pX458 (pSpCas9(BB)-2A-GFP) and either pEASY-HDR (FIX with 400 bp homologous arms) or ssODN-HDR (120 nucleotide donors carrying Y381D or Y381S mutations) *via* hydrodynamic tail vein injection. Survival after tail-clip challenge improved from 38% untreated mice to 86% in the Cas9/donor plasmid group. Sequencing confirmed 0.56% correction of *F9* alleles, with no significant changes in aspartate transaminase (AST) and Alanine transaminase (ALT) levels compared to controls (Figure 1). When adenoviral vectors (AdvCas9 and AdvG/T) were employed, correction efficiency increased to 5.5%, but clotting activity was not restored, and hepatic toxicity was observed (Guan et al., 2016).

Subsequent studies explored the integration of clotting factors FIX and FVIII at their native genomic loci. Depending on mutation type and location, either full-length cDNA can be integrated, or mutated coding regions can be replaced. Ohmori et al. (2017) created a hemophilia B mouse model by disrupting exon 8 using AAV8-CRISPR/Cas9. Mutation-specific gene editing by HDR increased FIX levels, but greater haemostatic correction was achieved by integrating codon-optimised *F9* cDNA (exons 2–8) into intron 1 of mouse *F9* gene (Figures 1, 2). High dose administration of AAV8-SaCas9 (1 × 10^12^ vg) and AAV8-mFIX (3 × 10^12^ vg), elevated plasma level to 11.7%–39.6%, within 4–8 weeks (Table 1) (Ohmori et al., 2017).

Wang et al. (2019) applied a similar approach, integrating human *F9* exons 2 to 8 carrying the Padua mutation (R388L) into exon 2 of the murine *F9* gene. Co-administration of AAV8.SaCas9 (5 × 10^11^ vg per mouse) and AAV8.sgRNA3. hFIXco-Padua donor (5 × 10^12^ vg per mouse) in FIX-KO mice produced supraphysiological FIX activity (171.6% of normal) within 1 week, sustained through week 32. While the hyperactive Padua mutation increased the gene editing efficiency in hemophilia B mice (Wang et al., 2019). This strategy may not be suitable for patients with promoter or proximal 5′ end coding sequence mutations.

In Hemophilia A, about half of patients with severe disease carry large chromosomal rearrangements in the *F8* gene. The most common, intron 22 inversion, accounts for about 45% of severe HA cases and results from crossing-over between intron 22 of *F8* and a duplicated sequence located ∼500 kb telomeric to the *F8* gene. A second inversion, found in ∼2–5% of HA cases, occurs in intron 1 through homologous recombination between a 1,041 bp segment of intron 1 and an extragenic copy ∼140 kb telomeric to the *F8* gene. Both inversions arise from erroneous repair of DNA double-strand breaks *via* non-allelic homologous recombination (NAHR) (Figure 4) (Cumming, 2004; Faridi et al., 2012; Wu et al., 2016).

Luo et al. (2021) explored precise editing of the intron 1 inversion to rescue exons 2-26 of the *F8* gene. Since mice lack the intron 1 repeats found in humans, an exact inversion model could not be created. Instead, a hemophilia A mouse model was generated by excising a 5,129 bp fragment that included the promoter, exon 1, and a part of intron 1 of using CRISPR/Cas9. To rescue expression, a donor fragment containing the human alpha 1-antitrypsin (hAAT) promoter, 146 bp of exon 1 coding sequence, and a splice donor site before exon 2 was integrated *via* AAV8 delivery (Figure 4). This restored *F8* mRNA levels from undetectable to 7.9%. Hemostasis improved, as shown by reduced aPTT and better bleeding outcomes in tail-clip assays 1 month after editing. Notably, even low doses of AAV vectors (4 × 10^10^ VG donor + 8 × 10^10^ VG CRISPR/SaCas9) significantly improved survival after tail-clip challenge (Luo et al., 2021).

Park et al. (2015) corrected both intron 1 and 22 inversions in patient-derived iPSCs using CRISPR/Cas9. For intron 1, DSB were induced at two homologous sites, allowing repair through error-prone NHEJ. Eight of 120 colonies were corrected for the intron 1 inversion. For intron 22, DSB were induced outside the duplicated regions to avoid off-target effects, resulting in five corrected colonies out of 135. When differentiated into endothelial cells, corrected iPSCs expressed functional FVIII, unlike uncorrected controls. Transplantation of these cells into hemophilia A mice restored FVIII activity to ∼10%, compared to 3.3% in untreated mice (Figure 4) (Park et al., 2015).

Son et al. (2022) used iPSCs from hemophilia A patients (F8d-HA-iPSCs) and CRISPR-corrected iPSCs (F8c-HA-iPSCs). Differentiation into CD157+ endothelial cells showed higher FVIII activity in corrected cells (Figure 3). Transplantation of 4 × 10^6^ corrected endothelial cells into hemophilia A mice reduced bleeding episodes and improved survival, with 90% of treated mice surviving after 3 months. The same study also generated 3D liver organoids by combining corrected endothelial cells with hepatocytes and mesenchymal stromal cells. Transplantation of ∼2,800 organoids achieved similar therapeutic benefits, including sustained FVIII expression, reduced bleeding and improved survival (Son et al., 2022).

Alternatively, Hu et al. (2023) corrected the intron 1 inversion by integrating a donor fragment containing the hAAT promoter and 146 bp of exon 1 before exon 2. This was achieved by nucleofecting iPSCs with plasmids encoding CRISPR/Cas9 complex, guide RNA, and donor template. The hepatocyte-like cells derived from corrected iPSCs showed *F8* transcripts detected by RT-PCR, hFVIII antigen detected by ELISA, and FVIII clotting activity detected by aPTT assay (Hu et al., 2023).

### 5.2 Integration in the albumin locus

Beyond editing native factor loci, researchers have also explored genomic safe harbours to achieve stable and durable transgene expression. The albumin locus is considered an ideal site for the integration of therapeutic transgenes due to its strong promoter activity and open chromatin, which supports high, liver-specific expression without disrupting albumin itself (Lu C. et al., 2022). The first exon of the albumin gene encodes a universal secretory signal peptide, making intron 1 a suitable integration site.

Wang Q. et al. (2020) integrated the human Padua FIX cDNA (exon 2 to exon 8) into intron 1 of the albumin locus using AAV8.SpCas9 (9 × 10^10^ VG) and AAV8.sgRNA donor vectors (4.5 × 10^11^ VG) (Figure 2). In hemophilia B mice, FIX activity reached 40% of normal at 16 weeks post-injection and remained stable through week 48, even after two-thirds partial hepatectomy (Table 1) (Wang Q. et al., 2020).

Interestingly, Chen et al. (2019) targeted the final intron (intron 13) of the albumin gene to preserve albumin structure while enabling expression of BDD-FVIII (Figure 2). Delivery of AAV8-SaCas9-sgRNA and AAV8-BDD-F8 vectors (6 × 10^11^ VG) increased FVIII in a dose-dependent manner. The expression of FVIII was detectable within 2 weeks and reached ∼13% of normal activity by 2 months at higher vector doses (Table 1). Importantly, albumin expression remained unchanged in treated mice (Chen et al., 2019).

### 5.3 Other integration sites

Efforts to correct hemophilia through gene editing have also expanded to include alternative integration sites. These strategies aim to achieve stable, durable and liver-specific expression of therapeutic transgenes.

#### 5.3.1 ROSA26

Stephens et al. (2019) injected four-weeks-old R333Q hemophilia mice with Adenoviral vectors: Ad5-EF1α-mFIX (7.5 × 10^10^ VG) and Ad5-cas9-gRNA (2.5 × 10^10^ VG) to knock in mouse FIX at the ROSA26 locus (Figure 1). Stable plasma mFIX levels of 700–850 ng/mL were maintained between 49 and 161 days, compared to 200–350 ng/mL in controls. At 238 days post-injection, treated mice averaged 419 ng/mL *versus* 88.5 ng/mL, in the control group (Table 1). Plasma FIX activity was <1% in the Ad5-cas9 group but ∼50% in the Ad5-cas9-gRNA + Ad5-EF1α-mFIX group. Off-target integration occurred at chromosomes 3 - (RP24–555P13, Genebank ID AC115945.9) and chromosomes 5 - Gamma-Secretase-Activating Protein (GSAP) (NM_001359876). Strong immune responses were observed, including IgG antibodies against Cas9 and adenoviral particles (detected at days 35 and 189) and Cas9-specific T cells producing IFN-γ that persisted up to 210 days (Stephens et al., 2019). While adenoviral vectors enabled efficient gene editing, immune activation and toxicity remain major limitations.

#### 5.3.2 APOC3

Lee et al. (2022) investigated the apolipoprotein C3 (*APOC3*) locus, a transcriptionally active site in the liver, as an alternative integration site for coagulation factors. Using AAV-CjCas9 and AAV-hF9 (4 × 10^13^ vg/kg each), hFIX was integrated into *APOC3* in humanized mice. Six weeks post-transduction, factor concentrations reached 250 ng/μL in the Homology-independent targeted integration (HITI) group and 50 ng/mL in the AAV-trap donor group (Table 1). These results highlight *APOC3* as a strong endogenous expression system for therapeutic transgenes (Lee et al., 2022).

#### 5.3.3 AAVS1

The iPSCs derived from an 11-year-old hemophilia B patient carrying the *F9* mutation (c.676C>T, p.Arg226Trp) were corrected by integrating normal *F9* cDNA (1.5 kb) under the EF1α promoter into the *AAVS1* safe-harbour locus on chromosome 19. The iPSCs were corrected by electroporating the AAVS1-Cas9-sgRNA and AAVS1-EF1α-F9 cDNA-puromycin plasmids. Differentiated hepatocytes from corrected iPSCs expressed higher hFIX antigen (44.4 ± 4.0 ng/mL) and activity (5.0% ± 0.3%) compared to uncorrected hepatocytes (1.6 ± 0.004 ng/mL and 1.7% ± 0.1%) (Figure 3). This demonstrates the potential of *AAVS1* as a safe-harbour site for durable hFIX expression (Lyu et al., 2018).

#### 5.3.4 H11 locus

Similarly, iPSCs carrying multiple exon deletions (exon 8–22) in the *F8* gene were corrected by knocking in *F8 cDNA* under the EF1α promoter at the *H11* locus (*hipp11* gene on chromosome 11), a recognised safer harbour site (Figure 2) (Park et al., 2019). The *H11* locus is advantageous for gene therapy because of its open chromatin structure, presence of an enhancer, and capacity to accommodate large DNA constructs (Zhu et al., 2014). Correction was achieved by electroporation of the plasmids that encode FVIII under *EF1α* promoter, Cas9 ribonucleoprotein, and sgRNA. *F8 mRNA* transcripts and FVIII activity were detected in both corrected iPSCs and their differentiated endothelial cells, but not in uncorrected controls (Park et al., 2019).

### 5.4 Correction of deletion mutation

In addition to intron 1 and 22 inversions, hemophilia A can also result from small frameshift deletions and large exon deletions. One example is a 4 bp frameshift deletion (c.3167del CTGA) in exon 14 of *F8.* Hu et al. (2019), derived iPSCs from the urine epithelial cells of a patient diagnosed with this mutation. This mutation was corrected by deleting a 54 bp region around the mutation site using dual-sgRNA and single-stranded oligodeoxyribonucleotides (ssODN). Frameshift mutations in the B-domain, such as this 4 bp deletion, cause premature termination of FVIII translation. By designing ssODN to enable an in-frame deletion (multiples of three base pairs), the reading frame was restored (Figure 2). Endothelial progenitor cells differentiated from corrected iPSCs were transplanted into hemophilia A mice, resulting in improved survival and increased FVIII activity (12.74% and 14.09%) compared with untreated controls (Hu et al., 2019). Overall, CRISPR/Cas9-based strategies for Hemophilia have demonstrated remarkable progress, ranging from mutation-specific correction to safe-harbour integration of clotting factor transgenes. These approaches restored FIX or FVIII activity in preclinical models, improved survival, and in some cases achieved supraphysiological levels of clotting factors. Despite these advances, challenges remain, including variable efficiency across mutation types, risks of off-target integration, immune responses to viral vectors and the need for long-term safety validation. Collectively, these findings highlight CRISPR/Cas9 as a powerful platform for hemophilia gene editing, with ongoing refinements required to translate preclinical success into durable clinical benefit.

### 5.5 Base editing

Beyond deletion correction, base editing offers a precise strategy for repairing point mutations. Tonetto et al. demonstrated that hemophilia A, caused by point mutations, can be effectively repaired using catalytically dead Cas9 nickase through base editing (BE) and prime editing (PE) (Figure 2). In HEK293T cells transfected with an FVIII construct carrying p.R2166* and p.R2228Q mutations, BE/PE restored **∼**25% of wild-type FVIII expression. Then, Base editing was further evaluated in engineered blood outgrowth endothelial cells (BOECs) harbouring the FVIII IVS22 mutation. Lentiviral vectors expressing FVIII variants, combined with the ABE8e-NG editing system and mutation-specific gRNAs, increased rFVIII levels to 17.4 ± 0.6 ng/mL (p.R2166*) and 32.9 ± 2 ng/mL (p.R2228Q). aPTT times were reduced from 167.8 ± 6.7 s to 52.8 ± 0.8 s (p.R2166*) and from 151.9 ± 14.7 s to 42.5 ± 2.5 s (p.R2228Q). Adenine Base Editing (ABE8e-NG) was favoured over the PE system due to its smaller coding cassette, which is better suited for lentiviral delivery in patient-derived BOECs (Tonetto et al., 2024).

Hiramoto et al. (2023) applied base-editing to hemophilia B iPSCs carrying the c.947T>C; I316T mutation. Using cytidine base editors (CBE) consisting of the D10A nickase version of Cas9 fused to deaminases (PmCDA1 or APOBEC1), the mutation was corrected by converting C to T. Corrected iPSCs were then differentiated into hepatocyte-like cells that expressed FIX, and transplantation into immunodeficient mice confirmed functional activity (Figure 2) (Hiramoto et al., 2023). Despite these promising results, further evaluation of the BE and PE systems in *in vivo* models is necessary to assess their feasibility and potential therapeutic application.

Together, these studies highlight complementary strategies for correcting hemophilia mutations. ssODN mediated deletion repair restores reading frames in case of small frame-shift mutations, while base editing directly corrects point mutations without introducing DSBs. Both approaches demonstrate functional rescue in cells and animal models, though further evaluation in *in vivo* systems is needed to establish long-term safety and therapeutic potential.

## 6 Rebalancing therapy for hemophilia

Rebalancing therapy is a strategy that enhances blood clotting by inhibiting natural anticoagulant pathways, compensating for the deficiency of clotting factors in hemophilia. Since hemophilia disrupts the balance between procoagulant and anticoagulant factors, reducing anticoagulants such as antithrombin (AT) can restore coagulation activity (Chen et al., 2023).

An AAV-Lipid Nano particle (LNP) hybrid strategy has been explored for this purpose. LNPs deliver Cas9 mRNA and sgRNA to the cytosol, where Cas9 is synthesised and then enters the nucleus. However, LNPs cannot deliver donor DNA for *knock-in*, which limits their use. Conversely, using dual or triple AAVs can lead to transduction interference, reduced *knock-in* efficiency, and potential immune responses at high doses. The hybrid AAV-LNP strategy lowers the required dose, mitigating these issues (Lee et al., 2023; Han et al., 2023).

Antithrombin, encoded by the *Serpinc1* gene, is a negative regulator of thrombin generation and thus a promising target (Pasi et al., 2017). Han et al. (2022) showed that downregulating the mouse *SerpinC1* with LNP-CRISPR improved thrombin generation in Hemophilic mice. Mice treated with LNP-CRISPR-mAT (1.2 mg/kg, three doses at 2-week intervals) reached stable anti-thrombin (mAT) levels by 10 weeks. *SerpinC1* gene function was reduced by over 70%, leading to thrombin peaks up to 65% higher than wild type, reduced spontaneous bleeding, and no significant off-target effects or inflammation. Notably, a single AAV-SpCas9 dose triggered strong anti-SpCas9 IgG, while repeated LNP-CRISPR-mAT dosing (3-doses) maintained minimal levels (Han et al., 2022).

**FIGURE 5:**
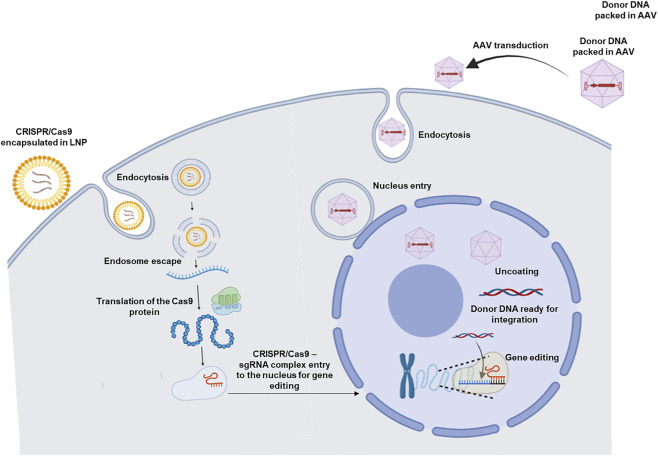
AAV-LNP gene editing for Hemophilia. LNPs deliver CRISPR/Cas9 mRNA and sgRNA, which leads to Cas9-sgRNA complex formation and enters the nucleus. AAV donor is delivered first to allow donor template delivery before DSB formation. AAV-LNP enables targeted gene editing to restore clotting factor expression. Image created using BioRender.

Subsequent work knocked *hF9* into *SerpinC1* exon 3 using LNPs (Cas9 mRNA + sgRNA) with an AAV donor (Figure 5). In *F9* mutant mice, AAV donor (2 × 10^13^ vg/kg) was administered before LNP-CRISPR to allow donor template delivery before DSB formation. Plasma FIX levels reached 1,000 ng/mL at 10 weeks and 500–700 ng/mL at 20 weeks, with no evidence of inflammation, liver toxicity or hepatocellular carcinoma after 26 months (Table 1) (Lee et al., 2023). Further improvements came with ionizable lipid 244-cis LNP combined with lower AAV dose. This LNP achieved 96.5% encapsulation efficiency and delivered mRNA effectively. AST and ALT levels were lower than those observed with ALC-0315 and SM-102 ionizable lipids used in COVID-19 vaccines. A single administration of AAV8-hF8-BDD (1 × 10^12^ vg/kg) plus LNP-CRISPR (1.5 mpk) reduced antithrombin (AT) concentrations by 30% and produced average hFVIII levels of 25 ng/mL in liver lysates (Figure 5) (Han et al., 2023). Overall, the CRISPR-mediated re-balancing approach represents a promising mutation-independent method to restore hemostasis by targeting natural anticoagulant pathways. This approach circumvents the limitations of FVIII/FIX replacement and offers the potential for durable therapeutic benefit. However, the inherent risk of thrombosis underscores the need for rigorous long-term safety evaluation before clinical translation.

## 7 Cellular context and durability of liver gene editing

Clinical experience with AAV-mediated liver gene transfer has consistently shown more durable expression in hemophilia B than hemophilia A. This disparity reflects not only vector tropism and native cell biology, but also intrinsic differences between FIX and FVIII as transgene products. FIX is a compact protein naturally synthesised in hepatocytes, which are efficiently transduced by AAV vectors. In contrast, FVIII is normally produced in Liver Sinusoidal Endothelial Cells (LSECs), a population not directly targeted by current designs, and its cDNA approaches the upper limit of AAV packaging capacity even in truncated B-domain deleted forms (Shahani et al., 2014) Beyond cell-type mismatch, FVIII imposes a substantial folding and secretory burden when expressed ectopically in hepatocytes, often triggering ER stress and unfolded protein responses that compromise durability. FVIII is also more immunogenic, with inhibitor formation and immune clearance contributing to variability in clinical outcomes (Chen et al., 2023; Kapelanski-Lamoureux et al., 2022). Although the adult liver is often described as quiescent, hepatocytes and LSECs undergo slow but continuous turnover. Animal studies suggest hepatocyte lifespans on the order of hundreds of days (Malato et al., 2011), while LSEC dynamics are less precisely defined but contribute actively to liver homeostasis (McConnell et al., 2023). Because AAV vectors persist mainly as episomes, transgene expression may decline as transduced hepatocytes or LSECs are replaced, particularly in tissues with high proliferative activity or in pediatric patients (Chuecos and Lagor, 2024). Thus, the relative success of FIX gene therapy reflects a convergence of favourable transcript size, protein stability, immunogenicity profile, and hepatocyte targeting, whereas FVIII presents multiple biological and technical hurdles. Genome-integrating approaches such as gene editing may ultimately provide more durable correction, as modifications are retained across successive cell generations.

## 8 Limitations: off-target editing, hepatotoxicity, and integration risks

Despite the promise of gene editing for hemophilia, several safety and efficacy concerns must be addressed before widespread clinical application. These limitations vary in their frequency, severity, and degree of clinical validation, and a balanced appraisal is essential for guiding future development. Gene editing for hemophilia shows promise but faces safety and efficacy challenges of varying magnitude. Rather than being uniformly prohibitive, these risks differ in their likelihood, detectability and clinical impact.

### 8.1 Off-target editing

CRISPR nucleases can introduce unintended mutations at sites with partial sequence similarity, including INDELS, large deletions, and chromosomal rearrangements (Wienert et al., 2019; Kosicki et al., 2018; Tsai and Keith Joung, 2016). While unbiased genome-wide assays have revealed such events, their frequency in the therapeutic context appears low. For example, Tonetto et al. (2024) corrected an *F8* point mutation with base editors, restoring FVIII expression and reporting limited off-target activity, though bystander edits remain a concern (Tonetto et al., 2024). Similarly, Wang Q. et al. (2020) found undetectable INDELs at predicted sites using targeted sequencing but cautioned that rare or complex events may escape detection (Wang Q. et al., 2020). Thus, off-target editing is a manageable but non-negligible risk, requiring comprehensive genome-wide evaluation in preclinical and clinical settings.

### 8.2 Hepatotoxicity and immune responses

AAV vectors, the main delivery system for liver editing, present a more immediate and clinically observed limitation. Dose-dependent hepatotoxicity, transaminitis, and immune-mediated loss of therapeutic efficacy have been repeatedly documented in AAV-FVIII trials (Ozelo et al., 2022; Mura et al., 2024; Greig et al., 2024; Dalwadi et al., 2021). Elevated ALT and AST levels often necessitate prolonged corticosteroid treatment to sustain transgene expression (Ozelo et al., 2022). At high doses, capsid-specific T-cell responses can eliminate transduced hepatocytes, directly reducing therapeutic benefit, leading to declines in FVIII or FIX levels (Ozelo et al., 2022; Mura et al., 2024). These events are clinically significant and recurrent, underscoring the need for careful dose selection, immune monitoring and improved vector engineering.

### 8.3 Integration risks

Although AAV genomes are primarily episomal, studies in non-human primates and human hepatocytes demonstrate integration at DSB. The long-term oncogenic potential of such events remains largely theoretical, with no clear evidence of malignancy in clinical hemophilia trials to date (Dalwadi et al., 2021; Greig et al., 2024). Nevertheless, such integration risks warrant long-term surveillance and mechanistic studies.

Among these limitations, hepatotoxicity and immune responses represent the most immediate barriers observed in clinical trials, while off-target edits and integration risks are less frequent but demand rigorous monitoring due to their potential consequences. Addressing these challenges will require a combination of improved nuclease specificity, safer vector designs, and long-term follow-up to ensure durable and safe correction.

## 9 Translational barriers to clinical application of gene editing for hemophilia

The clinical translation of gene editing for hemophilia is constrained by a complex interplay of biological, technical, economic and ethical challenges. Pre-existing immunity to Cas9 nucleases remains a major concern, as antibodies and memory T cells against *Streptococcus pyogenes* and *Staphylococcus aureus* Cas9 can eliminate edited cells, reduce efficacy and trigger inflammatory toxicity (Greig et al., 2024; Dalwadi et al., 2021). This immunogenicity not only limits initial dosing, but also complicates redosing strategies, particularly when combined with innate immune activation from viral or non-viral delivery systems. At the genomic level, the unusually large (∼186 kb) and repetitive *F8* locus (Zimmermann et al., 2010; Gitschier et al., 1984), often disrupted by intron 22 and intron 1 inversions, pose unique difficulties for guide RNA design and precise correction. These structural complexities magnify the limitations of current editors and interact with hepatocyte DNA repair biology, where reliance on non-homologous end joining over homology-directed repair restricts precise sequence correction and raises concerns about long-term durability, especially in pediatric patients with proliferating hepatocytes (Simoni et al., 2024; VanLith et al., 2019). This has led to the development of NHEJ-compatible approaches or DSB-free methods, such as base editing and prime editing.

However, editor-specific risks further highlight the translational tension between innovation and practicality. Base editors avoid DSBs, but introduce unintended edits within the editing window and guide- independent deamination, (Park and Beal, 2019; Jeong et al., 2020), while prime editors offer flexibility but suffer from low *in vivo* efficiency, pegRNA instability, large cargo size exceeding AAV limits, and risks of unintended insertions or deletions (Chen and Liu, 2023; Habib et al., 2022; Lu Y. et al., 2022; Anzalone et al., 2019; Mu et al., 2024). These challenges are compounded by delivery barriers: large CRISPR components remain difficult to package and administer efficiently, and both AAV and lipid nanoparticles face limitations in stability, dose-limiting toxicities and innate immune activation (Wang D. et al., 2020; Li et al., 2018). Delivery thus represents a central bottleneck, where immunity editor size and cost converge. Cost considerations themselves are substantial, as large-scale GMP vector manufacturing and extensive release testing drive expenses, particularly when dual vector systems or complex analytical characterisations are required. Although ongoing improvements in vector yield, potency, and delivery efficiency may reduce costs, these must be weighed against the very cumulative lifetime costs of conventional factor replacement therapy, which gene-based strategies have the potential to offset (Andaluz et al., 2025; Teodoro et al., 2024).

Finally, ethical considerations remain paramount. Long-term follow-up is essential to monitor risks such as unintentional germline exposure and the durability of editing effects in pediatric patients, while broader concerns include equitable access, informed consent in vulnerable populations and the societal implications of permanent genomic changes. (Ormond et al., 2017; Kohn et al., 2016; Muro et al., 2019). Taken together, these barriers underscore that translational success will require more than incremental improvements in editing efficiency. Progress will depend on high-fidelity editors, immune-orthogonal delivery systems, scalable manufacturing solutions, and enhanced monitoring tools, alongside careful patient selection and ethical oversight.

## 10 Conclusion

Recent advances in clinical gene therapy trials and preclinical genome-editing studies are paving the way for translating gene-editing treatments to hemophilia. Preclinical work in animal models and *in vitro* systems has shown that CRISPR/Cas9 or other nuclease-based correction of *F8* or *F9* can restore clotting function (Chen et al., 2019; Wang et al., 2019; Guan et al., 2016). Key priorities for clinical progress include improving on-target efficiency, reducing off-target effects, optimizing *in vivo* delivery methods, and confirming long-term safety in large-animal models. These goals are supported by advances in targeted gene insertion and repair, such as enhanced DNA-repair pathway modulation and improved donor design (Zhang et al., 2024). Two first-in-human trials mark the early clinical translation of gene editing in hemophilia. REGV131-LNP1265, an *in vivo* CRISPR/Cas9 therapy designed to insert a functional *F9* sequence, is under evaluation in adults with hemophilia B (NCT06379789) (https://clinicaltrials.gov/study/NCT06379789). BE-101, an autologous CRISPR-engineered B-cell therapy producing persistent FIX without conditioning, is being tested in the BeCoMe-9 trial (NCT06611436) (https://clinicaltrials.gov/study/NCT06611436). Clinical success in other monogenic blood disorders provides a strong translational context, as *ex vivo* CRISPR-edited autologous HSC therapies such as EDIT-301 and exa-cel (CTX001) have achieved sustained efficacy and high editing efficiency in sickle cell disease and β-thalassemia (Hanna et al., 2023; Frangoul et al., 2021). These precedents inform regulatory, manufacturing, and monitoring strategies applicable to hemophilia.

Importantly, the rationale for pursuing gene editing in hemophilia B must be distinguished from the progress already achieved with AAV-based gene therapy. FIX expression after AAV delivery has been relatively stable in clinical trials, and long-term follow-up suggests durable benefit in many patients. However, AAV approaches remain limited by vector dose requirements, hepatotoxicity, and pre-existing immunity to capsids, which restrict patient eligibility and complicate re-dosing. In contrast, gene editing offers the potential for permanent correction at the endogenous locus, thereby avoiding episomal loss, reducing variability in expression and enabling therapeutic benefit even in pediatric patients. Moreover, editing strategies may allow correction of diverse pathogenic variants rather than relying on transgene addition, and could be adapted to immune orthogonal delivery platforms that expand patient access. These theoretical advantages must be balanced against current uncertainties, including off-target risks, delivery efficiency, and long-term safety, which remain to be resolved in ongoing trials. By enabling durable locus-specific correction editing strategies hold promise to shift hemophilia care from lifelong management or transgene supplementation toward a one-time curative intervention.
